# Exposure to low doses of pesticides induces an immune response and the production of nitric oxide in honeybees

**DOI:** 10.1038/s41598-021-86293-0

**Published:** 2021-03-25

**Authors:** Merle T. Bartling, Susanne Thümecke, José Herrera Russert, Andreas Vilcinskas, Kwang-Zin Lee

**Affiliations:** 1 Institute for Insect Biotechnology, Justus Liebig University of Giessen, Heinrich Buff Ring 26-32, 35392 Giessen, Germany; 2 Fraunhofer Institute for Molecular Biology and Applied Ecology, Ohlebergsweg 12, 35394 Giessen, Germany

**Keywords:** Agroecology, Entomology, Biodiversity

## Abstract

Honeybees are essential pollinators of many agricultural crops and wild plants. However, the number of managed bee colonies has declined in some regions of the world over the last few decades, probably caused by a combination of factors including parasites, pathogens and pesticides. Exposure to these diverse biotic and abiotic stressors is likely to trigger immune responses and stress pathways that affect the health of individual honeybees and hence their contribution to colony survival. We therefore investigated the effects of an orally administered bacterial pathogen (*Pseudomonas entomophila*) and low-dose xenobiotic pesticides on honeybee survival and intestinal immune responses. We observed stressor-dependent effects on the mean lifespan, along with the induction of genes encoding the antimicrobial peptide abaecin and the detoxification factor cytochrome P450 monooxygenase CYP9E2. The pesticides also triggered the immediate induction of a nitric oxide synthase gene followed by the delayed upregulation of catalase, which was not observed in response to the pathogen. Honeybees therefore appear to produce nitric oxide as a specific defense response when exposed to xenobiotic stimuli. The immunity-related and stress-response genes we tested may provide useful stressor-dependent markers for ecotoxicological assessment in honeybee colonies.

## Introduction

Honeybees are pollinators that provide essential services in the maintenance of wild ecosystems, but they also ensure the stability of agricultural systems by securing crop yields that depend on insect pollination^1^. According to the Intergovernmental Science-Policy Platform on Biodiversity and Ecosystem Services (IPBES), managed bee colonies in Western Europe and North America have suffered high losses in the last few decades, raising concern in the scientific community, agricultural industry, and among the general public^2^. Although there is little or no information for many regions, available data show that the loss of honeybees is accompanied by the general decline of insect diversity and abundance^3,4^. The contributory factors are not fully understood^5,6^ but are likely to include biotic stressors (parasites and pathogens), abiotic stressors such as exposure to agrochemicals, and nutritional deficits caused by agricultural monoculture^7–9^. For example, exposure to sublethal doses of certain insecticides, including neonicotinoids, may affect physiology, development, behavior and reproduction, and may eventually cause a decline in honeybee populations^10–15^. The effects of low-dose neonicotinoids include impaired learning and homing behavior^16–20^, and greater susceptibility to pathogens such as microsporidians (*Nosema* spp.)^21–24^, deformed wing virus (DWV)^25^, and black queen cell virus (BQCV)^24^.

Although biotic and abiotic stressors may not immediately affect the survival of exposed individuals, they challenge the immune system and impair general fitness^7,10,26^. Honeybees are social insects, and may therefore compensate for their comparably small repertoire of immunity-related genes^27,28^ and cellular immune responses^29^ by developing behavioral mechanisms that limit intoxication via the avoidance and dilution of certain food sources, and the co-cultivation of beneficial microbes synergistically supporting the detoxification of plant metabolites^30^. However, general stress responses are evolutionarily conserved in eukaryotes, and many components discovered in model insects are therefore found in honeybees. For example, as in most insects, immunity in *Drosophila melanogaster* is mediated by larval plasmatocytes and adult hemocytes that are responsible for phagocytosis, autophagy^31,32^ and the secretion of small effector and signaling molecules known as antimicrobial peptides (AMPs) ^33,34^. Although AMP secretion is triggered by NF-κB signaling following the recognition of pathogen-related molecular patterns (PAMPs) such as bacterial lipopolysaccharides (LPS) and peptidoglycans, AMPs also protect insects from xenobiotics and are thought to modulate both the innate and adaptive immune responses^35^. Free radicals, in addition to their signaling^36^ and antioxidant functions^37^, also act as effectors in eukaryotic biotic and abiotic stress responses. They are produced by enzymes like dual oxygenase (DUOX), which generates reactive oxygen species (ROS) such as superoxide (^•^O^−^_2_), and nitric oxide synthase (NOS), which generates reactive nitrogen species (RNS) such as nitric oxide (NO). However, the accumulation of large quantities of free radicals can be cytotoxic, and their prolonged activity must be suppressed to avoid damage to host cells. This is achieved by the delayed expression of enzymes such as catalase, which can detoxify radicals and reactive species such as peroxynitrite^38^ or hydrogen peroxide^39,40^, and UDP-glucuronosyltransferases (UGTs), which form water-soluble glucuronides from those toxic and reactive compounds^41^.

To determine whether free radicals are involved in the intestinal immune response to xenobiotic agents and to identify stressor-dependent responses, we evaluated the effect of biotic and abiotic stressors on the immune response and stress-related signaling in the honeybee gut. We exposed adult workers to low doses of either an entomopathogenic bacterium or one of four different pesticides (two fungicides, one insecticide, and one herbicide) under laboratory conditions representing field-realistic concentrations. We then analyzed the expression of candidate genes encoding key immunity-related effectors such as AMPs, cytochrome P450 monooxygenases (CYPs), and enzymes such as catalase and UGTs that generate and detoxify free radicals.

## Results

### The lifespan of honeybees is affected in a stressor-dependent manner

**Figure 1:**
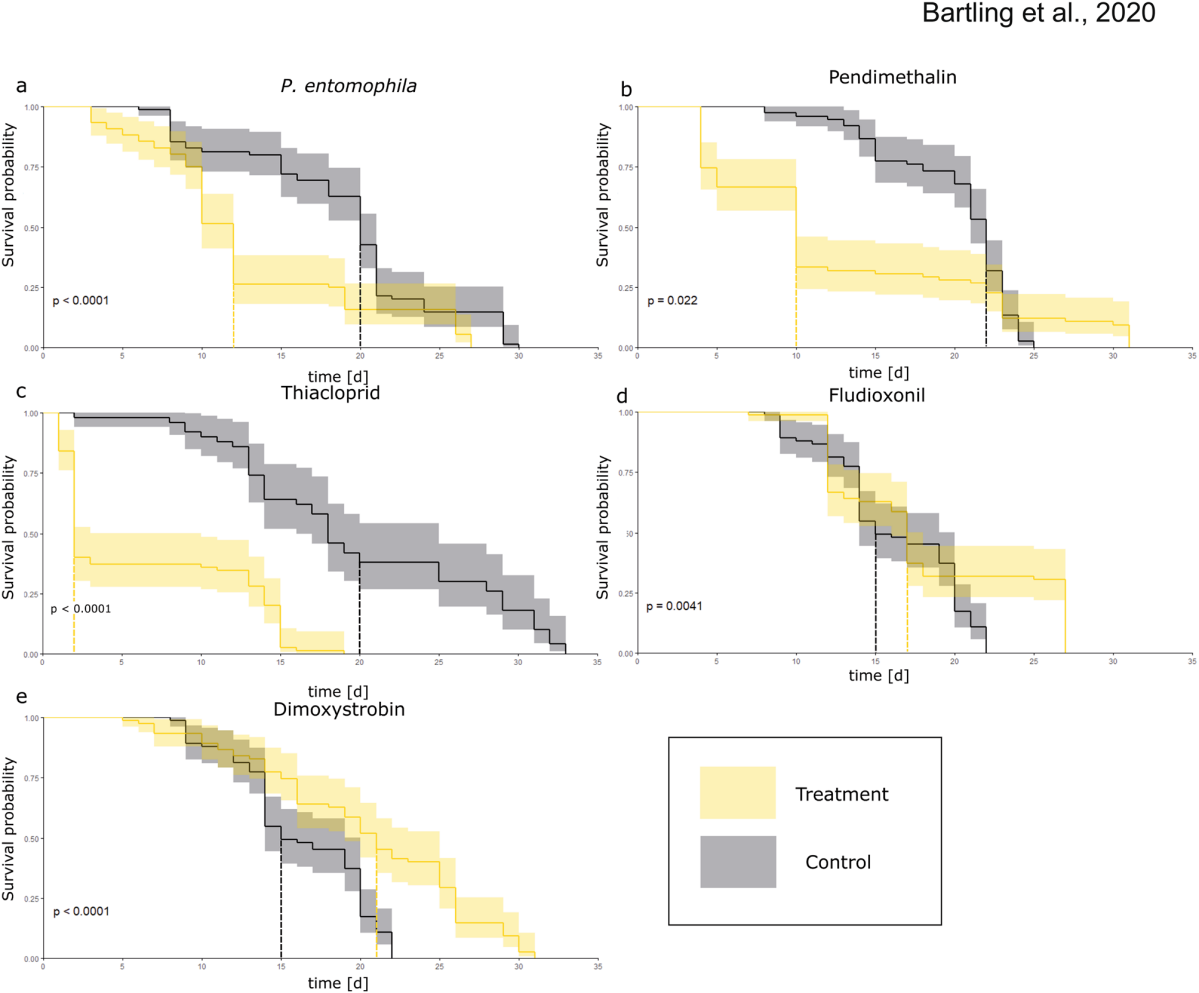
Survival rates over time after exposure to *P. entomophila* (biotic stressor) and low-dose abiotic stressors. Mean survival of *Apis mellifera* showing the effects of experimental supplements (yellow) compared to the control diet (black) over time. (**a**) *P. entomophila* (OD_600_ = 50). (**b**) Pendimethalin. (**c**) Thiacloprid. (**d**) Fludioxonil. (**e**) Dimoxystrobin. Mortality was recorded every day, with at least three biological and three technical replicas tested per condition. The level of significance is shown as *p* values. Kaplan–Meier estimators were derived for each experiment and plotted in *R* (R package version 3.2-7; https://CRAN.R-project.org/package=survival).

We investigated the effect of entomopathogens and pesticides on the lifespan of honeybees by supplementing the standard feeding solution with the bacterium *Pseudomonas entomophila*, the herbicide pendimethalin, the insecticide thiacloprid, or one of two fungicides (fludioxonil and dimoxystrobin). Honeybees orally infected with *P. entomophila* generally died sooner than controls fed on sugar syrup (Fig. 1). In six of the nine assays, we observed significant differences between the infected group and control (Fig. S1a) with mean LT_50_ values of 10 and 20 days, respectively (Fig. 1a). A similar effect was observed for honeybees exposed to pendimethalin, with mean LT_50_ values of 10 days in the exposed group compared to 23 days in the control (Fig. 1b). However, some exposed individuals significantly outlived the controls, surviving up to 31 days after treatment. As expected, thiacloprid also reduced the honeybee lifespan, with LT_50_ values of 14 days compared to 20 days for the controls (Fig. 1c). In contrast, exposure to the non-systemic fungicide fludioxonil had a complex effect, with some assays indicating that the treated group survived for a significantly shorter period (LT_50_ = 13 days) than controls (LT_50_ = 18 days) and others showing a significantly longer survival period (LT_50_ = 27 days) than controls (Fig. S1d), leading to a non-significant difference in the LT_50_ between the fludioxonil-treated and control groups overall (Fig. 1d). Interestingly, the strobilurin fungicide dimoxystrobin appeared to extend the honeybee lifespan from a mean LT_50_ of 15 days in the control to 21 days in the treated group (Fig. 1e).

### Immunity-related and stress-related genes are induced by pesticide challenge

**Figure 2:**
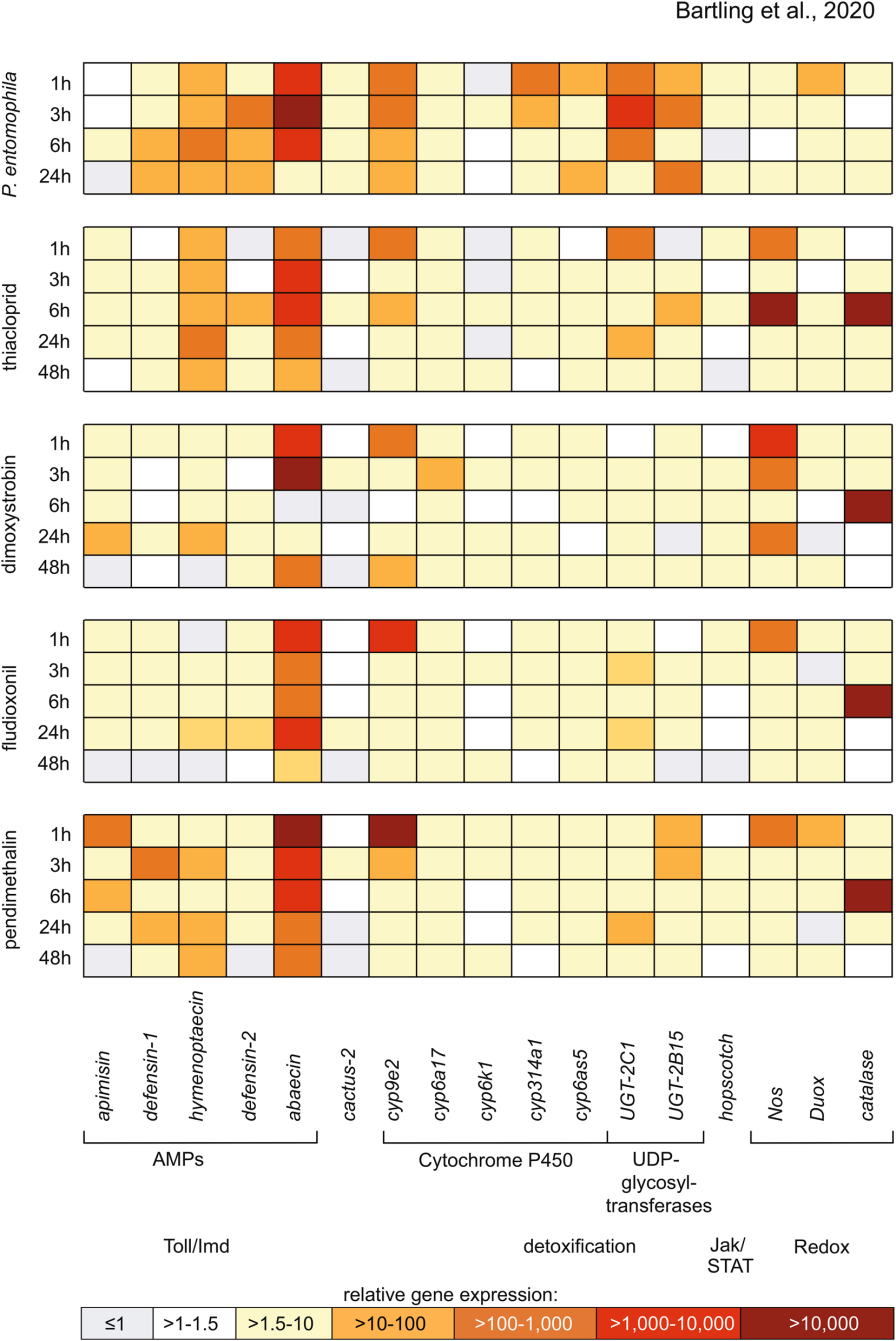
Gene expression heat map for immunity-related and stress-dependent genes. Analysis of immunity-related and stress-dependent candidate gene expression in the midgut of *Apis mellifera* after feeding with *P. entomophila* or one of four different xenobiotics. Gene expression was determined after 1, 3, 6, 24 and (for pesticide treatments) 48 h. Each square represents the mean relative expression of three biological and three technical replicates. Expression is color coded for rages from 0 to > 10,000 as indicated by the scale. Upregulation of gene expression was defined as > 1.5 (yellow to dark red on the scale). The figure was created with Microsoft Office 2010 using Excel and PowerPoint.

To evaluate the effects of low pesticide doses on the immunity and stress responses of honeybees, we analyzed the expression of 17 marker genes encoding AMPs, detoxification enzymes and redox factors by qRT-PCR, using samples of bee gut tissues dissected 1, 3, 6, 24 and 48 h after pesticide ingestion. Similarly, marker gene expression was tested 1, 3, 6 and 24 h post-infection with *P. entomophila* (Fig. 2). We observed the induction of AMPs (abaecin, apisimin, defensins 1 and 2, and hymenoptaecin) in response to most of the stressors at most of the sampling time points. Although the overall induction was weak to moderately high (1.5-fold to 100-fold), the gene encoding the AMP abaecin was consistently and strongly induced (> 10,000-fold) in response to the bacteria and the pesticides, peaking at the early time points (1–3 h). The gene encoding the AMP hymenoptaecin was also induced (1.5-fold to 1000-fold), with the strongest induction (up to 1000-fold) in response to thiacloprid and *P. entomophila*. Interestingly, a third AMP gene (encoding apisimin) was repressed or weakly induced (1.5-fold to tenfold) in response to *P. entomophila* but moderately induced (≤ 1000-fold) in response to pendimethalin at the earlier time points. The inhibitory Toll pathway gene *cactus-2* showed weak induction at some individual time points in response to the pesticides, mostly with values below 1.5-fold. However, *cactus-2* was weakly (≤ tenfold) but consistently induced throughout the time course in response to *P. entomophila*.

Several CYP genes were weakly (≤ tenfold) or moderately induced (≤ 1000-fold), but *cyp9e2* was strongly induced (up to > 10,000-fold) by *P. entomophila* and the pesticides at the early time points, indicating a role in the immediate response to these stressors. Two genes encoding UDP-glucuronosyltransferases (UGTs) were strongly induced (≤ 10,000-fold) by *P. entomophila* but only moderately induced (generally ≤ 1,000-fold) by the pesticides. The *hopscotch* gene encoding a tyrosine kinase in the JAK/STAT pathway was only weakly induced (≤ tenfold) regardless of the treatment. The *Nos* gene was moderately (≤ 1000-fold) or strongly (≤ 10,000-fold) induced by all the pesticides after 1 h, but only weakly (≤ tenfold) induced in response to *P. entomophila*. Similarly, the gene encoding catalase was strongly upregulated (> 10,000-fold) after 6 h, but only in response to the pesticides. The *Duox* gene encoding dual oxidase was minimally induced by all the stressors.

The analysis of gene expression therefore revealed three sets of genes strongly induced by the stressors we tested—one set of genes (principally *abaecin* and *cyp9e2*) induced by the pesticides and the bacterial pathogen, another (principally the UGT genes) induced strongly by the pathogen but only weakly or moderately by the pesticides, and a third (principally *Nos* and *catalase*) induced by the pesticides alone, with a significant delay between the immediate expression of *Nos* and the subsequent expression of *catalase*.

### Free radicals show distinct accumulation profiles in the honeybee gut

**Figure 3:**
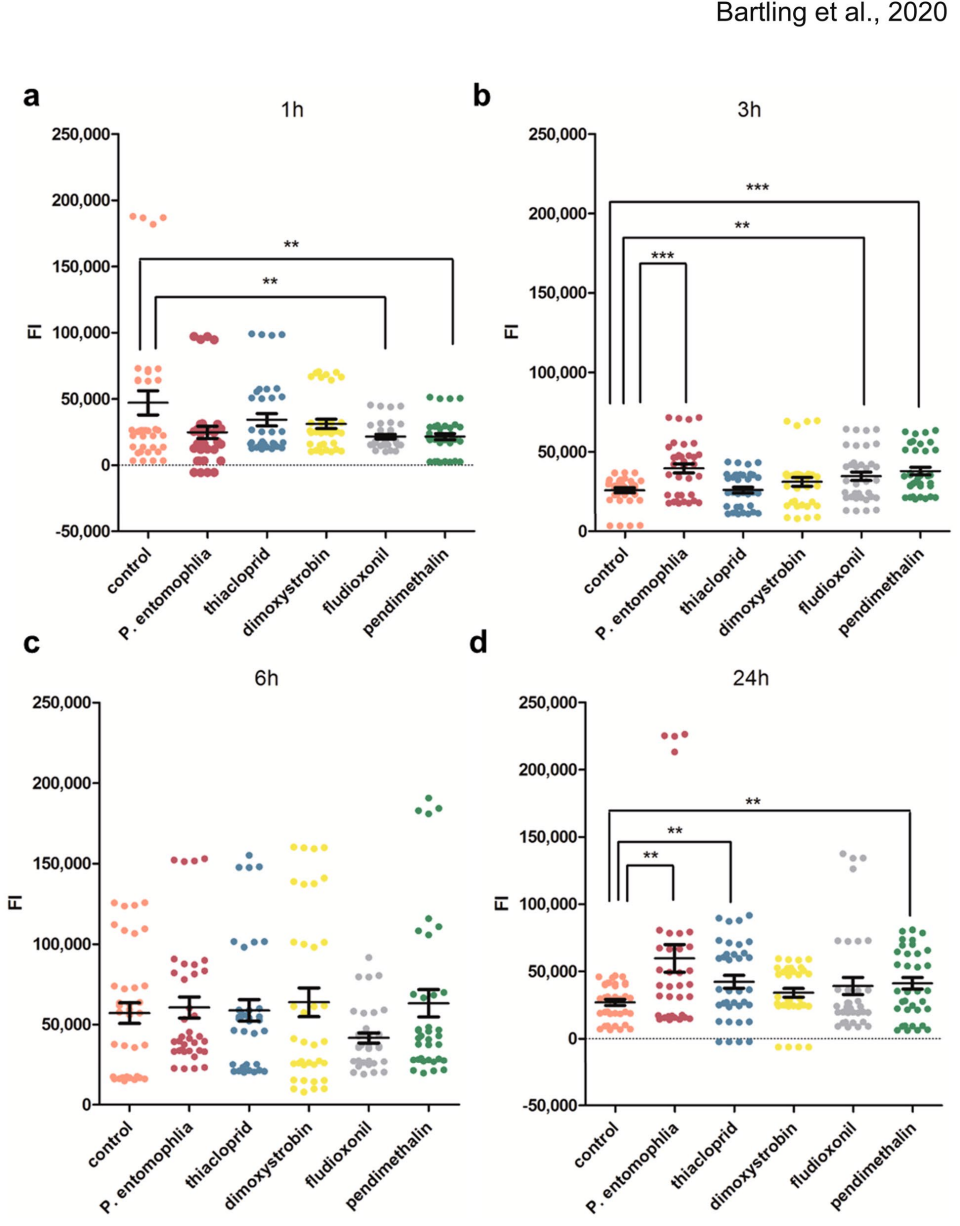
Quantification of reactive oxygen species in the midgut of *Apis mellifera*. The production of reactive oxygen species in midgut tissues was measured at four different time points after challenge using the general oxidative stress indicator CM-H_2_DCFDA. After 1 h, fluorescence intensity (FI) was significantly lower for fludioxonil and pendimethalin compared to the controls. After 3 h, an increase in FI was detected for *P. entomophila*, pendimethalin and fludioxonil. After 6 h, no significant changes were detected. An increase in FI was observed for *P. entomophila*, thiacloprid and pendimethalin after 24 h. The Shapiro–Wilk test and Wilcoxon matched-pairs signed rank test were used for statistical evaluation. The level of significance is indicated by asterisks. The figure was created with GraphPad Prism version 9.0.0 for Windows, GraphPad Software, San Diego, California USA, www.graphpad.com.

The almost immediate upregulation of *Nos* by abiotic stress followed by the delayed induction of *catalase* suggested that free radicals may generally be involved in the stress response to the pesticide treatments. We therefore used a general indicator for oxidative stress, CM-H_2_DCFDA, to quantify the free radical levels at four time points after each pesticide treatment (and following exposure to *P. entomophila*) compared to untreated controls. After 1 h, the fluorescence signal was lower than the control in all five treatments, significantly so in the case of fludioxonil and pendimethalin (Fig. 3a). After 3 h, the fluorescence signal was significantly higher in the *P. entomophila* and pendimethalin treatments compared to the control, and we observed a smaller but still significant increase for the fludioxonil treatment (Fig. 3b). After 6 h, we observed no significant difference between the five treatments and untreated control (Fig. 3c). After 24 h, we once again observed significant differences between the control and the treatments with *P. entomophila*, thiacloprid and pendimethalin (Fig. 3d).

## Discussion

The impact of various biotic and abiotic stressors on honeybees is not well understood. We therefore tested the effect of one entomopathogen and low doses of four pesticides on honeybee survival as well as the expression of immunity-related and stress-response genes. Accordingly, we exposed adult worker bees orally to the entomopathogenic bacterium *P. entomophila*, the fungicides fludioxonil and dimoxystrobin, the herbicide pendimethalin, and the insecticidal neonicotinoid thiacloprid.

We found that survival depended on the individual stressors, but that the effect of the treatment was not always the same in replicate tests, arguably reflecting the different ages of the individual bees obtained from the same hive^42–45^. However, using bees from a single hive reduced the variability of the assays because the individuals faced the same environmental influences and shared the same resources, in contrast to bees from different hives. Nevertheless, the honeybee lifespan was reduced by the insecticide thiacloprid and, in the majority of tests, by the entomopathogenic bacterium and the herbicide pendimethalin. This is supported by previous ecotoxicological studies that showed a moderate to low effect of pendimethalin on honeybee health^46^. Interestingly, dimoxystrobin generally increased the honeybee lifespan, whereas fludioxonil showed different effects in different test replicates and the overall effect on survival was nonsignificant. We did not control for behavioral adaption regarding food selection and quality, so fungicide intoxication may have been compensated by natural detoxification^30^, or whether the reproduction compensated for individual losses, as previously suggested^47^. The oral route was used in this study to expose honeybees to low doses of each xenobiotic, resulting in pesticide concentrations similar to those previously found as residues in bee bread. Therefore, the shorter lifespan of honeybees exposed to pendimethalin and thiacloprid was not surprising, nor was it necessarily anticipated. Although thiacloprid targets the nicotinic acetylcholine receptor^48^, the effect of low doses on honeybee survival is inconclusive^24,49–51^. In contrast, the extended lifespan of insects in response to low doses of toxins and free radicals has been described as an evolutionary adaption (hormesis), boosting the expression of genes that protect cells from stress^52^. This may have contributed to the prolonged lifespan following exposure to dimoxystrobin. Despite the increased mortality caused by exposure to fungicides combined with insecticides^53^, the effects of single fungicides, including dimoxystrobin, on bee mortality have not been characterized in detail.

The analysis of gene expression levels provided insight into the effects of the entomopathogen and different xenobiotic stressors on the honeybee immune system. *P. entomophila* has previously been shown to induce an immune response in honeybees under laboratory conditions^54^. As expected, the bacterium was well suited as a representative biotic stressor, not only reducing the honeybee lifespan but also inducing many of the immunity-related and stress-dependent marker genes. *P. entomophila* induced most of the AMP genes, as well as *cyp9e2* and both *UGT-2C1* and *UGT-2B15*, but the redox-defense genes *Nos* and *Duox*, or the *catalase* gene were only weakly upregulated, even though the corresponding products are essential for antimicrobial activity^55^. The response to the entomopathogen differed qualitatively from the response to the pesticides, but all four pesticides induced similar responses indicating the activation of a conserved mechanism to counter the stress imposed by xenobiotics. We observed the strong induction of genes encoding the AMP abaecin, CYP9E2, NOS and catalase. The *hymenoptaecin* gene was strongly induced by *P. entomophila* and the insecticide and to a lesser extent by the other pesticides. Abaecin and hymenoptaecin were previously shown to work synergistically, with the combined antibacterial activity greater than the sum of each component’s activity when presented alone^35^. This may indicate a specific synergistic response to thiacloprid and *P. entomophila*, although the strong expression of *abaecin* in response to all treatments suggests that abaecin may play a universal, stressor-independent role in defense. The two main functions of AMPs are the recognition of pathogens via PAMPs such as LPS and peptidoglycans, and the metabolism of xenobiotics^56^. The stressor-independent induction of *abaecin* suggests that this AMP is involved in both activities.

Invertebrate humoral defense involves stressor recognition followed by elimination, facilitated by the activation of AMPs and the production of toxic superoxide anions and hydrogen peroxide^32,57^. Although the production and segregation of ROS and RNS primarily involves the hemocytes and fat body^58^, these reactive species are also known to confer antimicrobial activity in the gut epithelium^32,59^. Interestingly, *Duox* was only moderately upregulated in the gut (if at all) regardless of the stressor. In *D. melanogaster*, dual oxygenase is the most important factor in the initiation of an immune response against invading microbes^60,61^, and the neonicotinoid imidacloprid specifically interferes with this pathway^62^. In contrast, we found that *Nos* expression was strongly and immediately induced in response to the pesticides, peaking within 1–3 h in most cases. In the case of thiacloprid exposure, even stronger *Nos* induction was detected after 6 h, correlating with the *catalase* expression peak, and possibly indicating the specificity (hence higher toxicity) of the insecticide. The defense against xenobiotics therefore appears to activate RNS rather than ROS. Highly-reactive NO, produced by the oxidation of arginine to citrulline by NOS^63^, is considered a key effector in the defense responses of invertebrates by interacting with ROS such as superoxide anions and hydrogen peroxide^59^, as well as signaling for the induction of AMPs^64,65^. ROS and RNS intermediates react to form other cytotoxic compounds such as peroxynitrite with a synergistic mode of action^38,66^. Although the fluorescent dye CM-H_2_DCFDA generally indicated oxidative stress with the moderate accumulation of ROS after 3 h, the potential contribution of the gut microbiome cannot be ruled out, and the specific reactive molecules could not be identified. Further experiments are required to specifically detect the nitrogen-derived compounds we assume are responsible for the observed effect. The weak induction of *Nos* and *Duox* by the entomopathogen *P. entomophila* aligns with previous reports showing that this bacterium can inhibit *Duox* expression^54^, possibly reflecting an evolutionary strategy to inhibit ROS production based on uracil sensing^67^. It is unclear whether *P. entomophila* achieves the suppression of insect defenses by directly modulating redox-related genes that were not tested in our experiments, or indirectly by, for example, influencing the composition of the gut microbiome. Nevertheless, the upregulation of CYP and UGT genes suggests that a strong detoxification response is induced by the entomopathogen, indicating the presence of free radicals. Despite the weak upregulation of *Nos*, the production of NO by hemocytes to facilitate an immune response in the gut is nevertheless possible^68,69^.

ROS and RNS are useful as an immediate response against stressors, but their persistence is likely to damage host cells^70^. Accordingly, they are removed by protective antioxidant enzymes such as catalase and detoxification enzymes such as CYPs and UGTs, which bind molecular oxygen and other cytotoxic compounds to directly form non-toxic water or water-soluble products in insects and mammals^41,71^. We observed the strong upregulation of *catalase* following the induction of *Nos*, suggesting the role of catalase is to clear up RNS produced by NOS. Further experiments are required to measure free radical levels and to determine which compounds are removed by catalase. The *catalase* gene was moderately upregulated in response to *P. entomophila*, coinciding with the minimal induction of *Nos* and *Duox*. In contrast, *UGT-2C1* and *UGT-2B15* were induced more strongly by the entomopathogen than the pesticides. Most of the CYP genes were moderately induced, but *cyp9e2* was upregulated 100–10,000-fold after 1 h for all stressors, indicating a detoxification function that is not restricted to bacterial infections^72^. Indeed, CYP9E2 has been shown to metabolize thiacloprid efficiently in honeybees, whereas other CYPs cannot fulfil this function^73^. Moreover, a recent analysis of the sublethal effects of air pollution, a chemically complex stressor, also showed that *cyp9e2* was strongly upregulated in honeybee heart tissue^74^. Our data indicate that biotic and abiotic stressors induce the preferred expression of genes encoding UGTs and catalase, respectively, whereas CYP9E2 appears to fulfil a universal detoxification function. The ability of dimoxystrobin to disrupt the mitochondrial respiratory chain in fungi may explain the particularly strong induction of *cyp9e2* (> 10,000-fold) by this xenobiotic.

Interestingly, our gene expression data were not homogeneous at the various sampling time points, possibly reflecting the widely spaced sampling intervals but also the fact that our insects were collected from a working hive rather than synchronously bred in the laboratory. Age and developmental stage may influence the potency of stress responses and immunity in bees^43,44^. Regardless of the stressor, the primary stress response of eukaryotic cells relies on the immediate activation of defense signaling molecules such as ROS^75–77^. However, the production of these volatile compounds in response to xenobiotics, followed by their elimination, is a dynamic cell state that may also explain the results of our time course experiments. In addition, our gene expression data clearly indicate the induction of AMP genes in response to stress. In contrast to the increase in AMP gene expression we observed, previous studies in honeybees and masonbees showed that low doses of neonicotinoids cause the depletion of hemocytes, resulting in limited antimicrobial activity^78–80^. Moreover, we did not observe significant upregulation (> 1–10 for only single timepoints) of the Toll inhibitor *cactus-2* following xenobiotic exposure. Assumably, the weak induction still allowed an active immune response, a hypothesis that would require further studies with Toll pathway components. In contrast, RNA interference experiments have shown that clothianidin induces *relish*, encoding an inhibitor of the NF-κB signaling pathway, thus promoting the replication of DWV^81^. All these studies involved animals under controlled laboratory conditions, undoubtedly facilitating the collection of more homogenous data, but ruling out the environment as a potential factor responsible for the “natural priming” of the immune system. In contrast, we administered different stressors that, with the exception of thiacloprid, do not primarily target insects, using individuals collected directly from one hive. These insects therefore shared the same resources and were exposed to the same environmental conditions. Different studies of the same stressors have often reached dissimilar conclusions, probably influenced by factors that are not controlled in the experiment, such as the age of individual insects^82^. Similarly, the level of toxin residues in bees exposed to imidacloprid differed greatly between animals that were fed in groups of 10 and those fed individually^83^. Such deviations may explain the heterogeneity in some of our results (e.g., the replicates in the survival experiments) and confirm the significant effect of environmental conditions on honeybee health. Honeybees are continuously exposed to multiple anthropogenic stressors as well as natural changes that affect their resistance in ways that are not fully understood^5,84^.

We have shown that *P. entomophila* (a biotic stressor) and low doses of abiotic stressors induce specific immunity-related and stress-response genes in the intestine of adult honeybees, whereas other key candidate genes such as *Duox* and the JAK/STAT pathway component *hopscotch* do not appear to be involved. The key players in the detoxification of stressors that are not designed specifically to target honeybees are NOS, which promotes the release of NO, and the AMP abaecin. We also found that the entomopathogen *P. entomophila* strongly induces genes encoding UGTs but induces *Duox* only weakly. Our results therefore indicate that the intestinal immune response can differentiate between biotic and abiotic stressors, but distinct xenobiotic stressors appear to trigger the same host responses. Our study helps to unravel the molecular mechanisms underlying xenobiotic stress responses in the honeybee gut, revealing the activation of NOS in the intestinal epithelium, in turn triggering a NO-mediated defense response followed by the activation of catalase to minimize self-inflicted damage. The most responsive marker genes we tested, such as *cyp9e2* and *catalase*, may provide useful biomarkers for ecotoxicological assays in honeybee populations^85,86^.

## Methods

### Beekeeping

Western honeybees (*Apis mellifera carnica*, Pollmann 1879) were obtained from a beehive located near the Institute of Insect Biotechnology in Giessen, Germany (50° 34′ 05.8′′ N, 8° 40′ 18.6′′ E). For the survival experiments, the hive was opened and female bees of all ages were collected from the frames. For the immune response experiments, forager bees were collected from the hive entrance to ensure the immune system was already primed by their natural environment^87^.

### Feeding

The honeybees were fed on Apiinvert sugar syrup (Südzucker, Mannheim, Germany) supplemented with the entomopathogen *P. entomophila* (OD_600_ = 50), 129 µg/l thiacloprid, 94 µg/l dimoxystrobin, 357 µg/l fludioxonil or 53 µg/l pendimethalin (MilliporeSigma, St. Louis, MO, USA). The pesticide concentrations were based on the list of pesticide residues in bee bread according to the interim report of the German Bee Monitoring Project 2015 (DeBiMo 2015). The pesticides were compounded with sugar syrup and stored at 4 °C. Pendimethalin is not soluble in sugar syrup, so 0.1% dimethyl sulfoxide (DMSO, MilliporeSigma) was added to the diet, and also to the control diet of pure Apiinvert.

### Survival analysis

For each treatment, 25 honeybees were kept in a small ventilated plastic box (10 × 10 × 8 cm) with tap water and food provided ad libitum. To ensure a favorable environment, a pheromone stick (4–5 mm) containing queen mandibular pheromone (Phero Tech, Delta, Canada) was placed in the boxes. Every day, surviving bees were counted and provided with fresh food and water. Bees were kept in the dark at 26° C and 60% relative humidity. Survival assays were carried out three times per treatment and control.

The *R* libraries *survival* and *survminer*^88^ were used to fit Kaplan–Meier curves to the survival data and plot the statistical analysis and mean LT_50_ for Fig. 1. Statistical values for each single assay (Fig. S1) were determined using GraphPad Prism version 9.0.0 (GraphPad Software, La Jolla, CA, USA). Survival assays in which the control animals survived less than 15 days were not considered for statistical analysis. To determine significant differences between replicates, we carried out a log-rank (Mantel–Cox) test with subsequent Bonferroni correction.

### Analysis of gene expression

For each treatment and sampling time point, nine bees in three biological and three technical replicates were provided with 70 μl of sugar syrup. Fed animals were kept individually at 26 °C and 60% relative humidity in the dark. Guts were dissected 1, 3, 6, 24 and 48 h after feeding. Dissected midguts were placed in 2-ml screw cap tubes (Sarstedt, Nümbrecht, Germany) and homogenized using a FastPrep-24 homogenizer (MP Biomedicals, Santa Ana, CA, USA). RNA was extracted using TRIzol reagent according to the supplier’s instructions (Thermo Fisher Scientific, Waltham, MA, USA) and the RNA purity and quantity were measured using a NanoDrop spectrophotometer (VWR, Radnor, PA, USA). The RNA was reverse transcribed using the iScript system (Bio-Rad Laboratories, Hercules, CA, USA) according to the manufacturer’s instructions. Gene expression levels were determined by quantitative real-time PCR (qRT-PCR) using primers designed with Primer3 and BLAST (Table S1). The honeybee *eIF3-S8* gene encoding eukaryotic translation initiation factor 3 subunit C was used as the internal reference. Samples were amplified in 96-well plates on a StepOnePlus Real-Time PCR system (Applied Biosystems, Foster City, CA, USA) using PowerUp SYBR Green Master Mix (Thermo Fisher Scientific). Melting curves and primer tests were used to evaluate primer specificity. The cycling conditions are summarized in Table S1. Absolute expression ΔCt values were determined by normalization to the reference gene. The relative expression levels of candidate genes were calculated using the 2^−ΔΔCt^ method. Values > 1.5 were defined as an increase of gene expression. Gene expression data were analyzed using StepOne v2.3 (Applied Biosystems) and Microsoft Excel 2010 (Microsoft Corporation, Redmond, WA, USA). Comparisons between treatments were based on the means of three biological and three technical replicates and were used to generate a heat map.

### Quantification of ROS

For each treatment and sampling time point, nine bees in three biological and three technical replicates were provided with 70 μl of sugar syrup. Fed animals were kept individually at 26 °C and 60% humidity in the dark. Guts were dissected 1, 3, 6 and 24 h after feeding and stained for 20 min with 10 µM CM-H_2_DCFDA (Thermo Fisher Scientific) in PBS containing 2 mg/ml of the catalase inhibitor 3-amino-1,2,4-triazole (MilliporeSigma). Gut tissues were homogenized using a FastPrep-24 homogenizer and the fluorescence intensity was measured using the bottom optics of a CLARIOstar microplate reader (BMG Labtech, Ortenberg, Germany) with excitation at 485 nm, emission at 538 nm and a gain of 1800. Data were analyzed using Microsoft Excel 2010 and GraphPad Prism version 9.0.0.

## Supplementary Information

Supplementary Information 1.

Supplementary Information 2.
