# Supplementary material for: Exposure to low doses of pesticides induces an immune response and the production of nitric oxide in honeybees
Source: Sci Rep. 2021 Mar 25;11:6819. doi: 10.1038/s41598-021-86293-0 (PMC7994568; doi:10.1038/s41598-021-86293-0)

Exposure to low doses of pesticides induces an immune response and the production of nitric oxide in honeybees

Merle T. Bartling[a], Susanne Thümecke[a], José Herrera Russert[a], Andreas Vilcinskas[a,b], Kwang-Zin Lee[b,1]

[a]Institute for Insect Biotechnology, Justus Liebig University of Giessen, Heinrich Buff Ring 26-32, D−35392, Germany
[b]Fraunhofer Institute for Molecular Biology and Applied Ecology, Ohlebergsweg 12, D-35394 Giessen, Germany

[1]To whom correspondence may be addressed. Email kwang-zin.lee@ime.fraunhofer.de

Figure S1

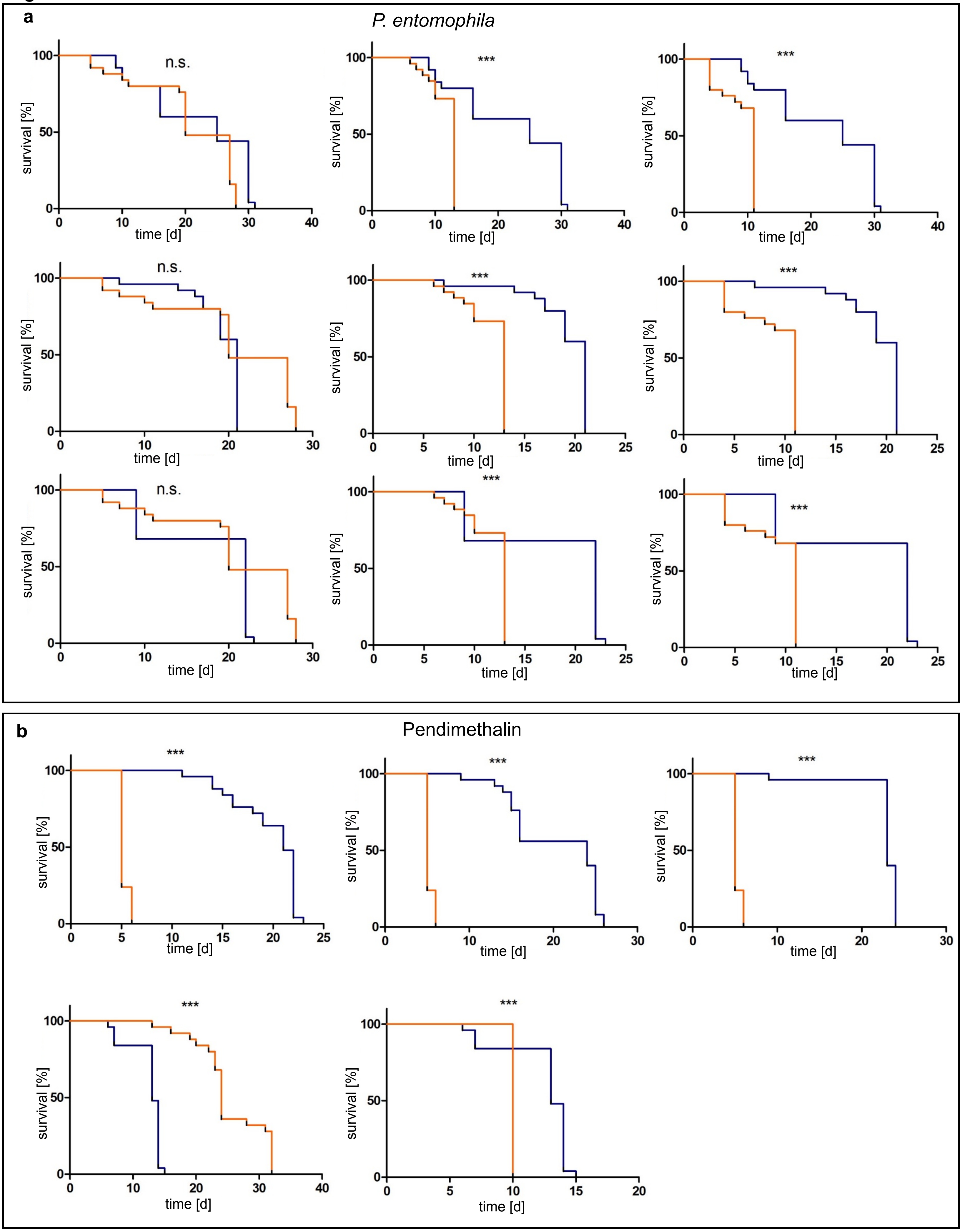

Exposure to low doses of pesticides induces an immune response and the production of nitric oxide in honeybees

Merle T. Bartling[a], Susanne Thümecke[a], José Herrera Russert[a], Andreas Vilcinskas[a,b], Kwang-Zin Lee[b,1]

[a]Institute for Insect Biotechnology, Justus Liebig University of Giessen, Heinrich Buff Ring 26-32, D−35392, Germany
[b]Fraunhofer Institute for Molecular Biology and Applied Ecology, Ohlebergsweg 12, D-35394 Giessen, Germany

[1]To whom correspondence may be addressed. Email kwang-zin.lee@ime.fraunhofer.de

Figure S1

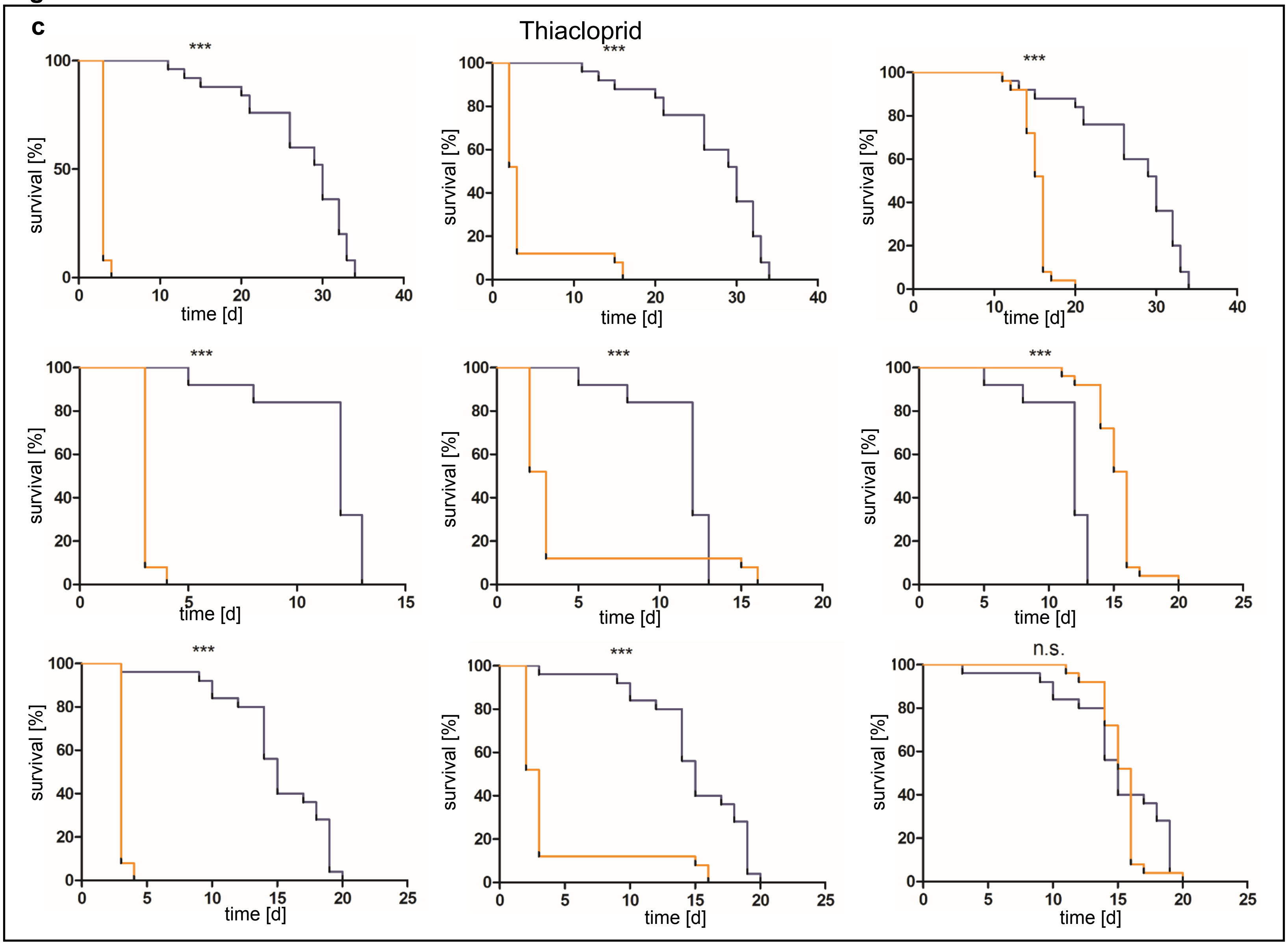

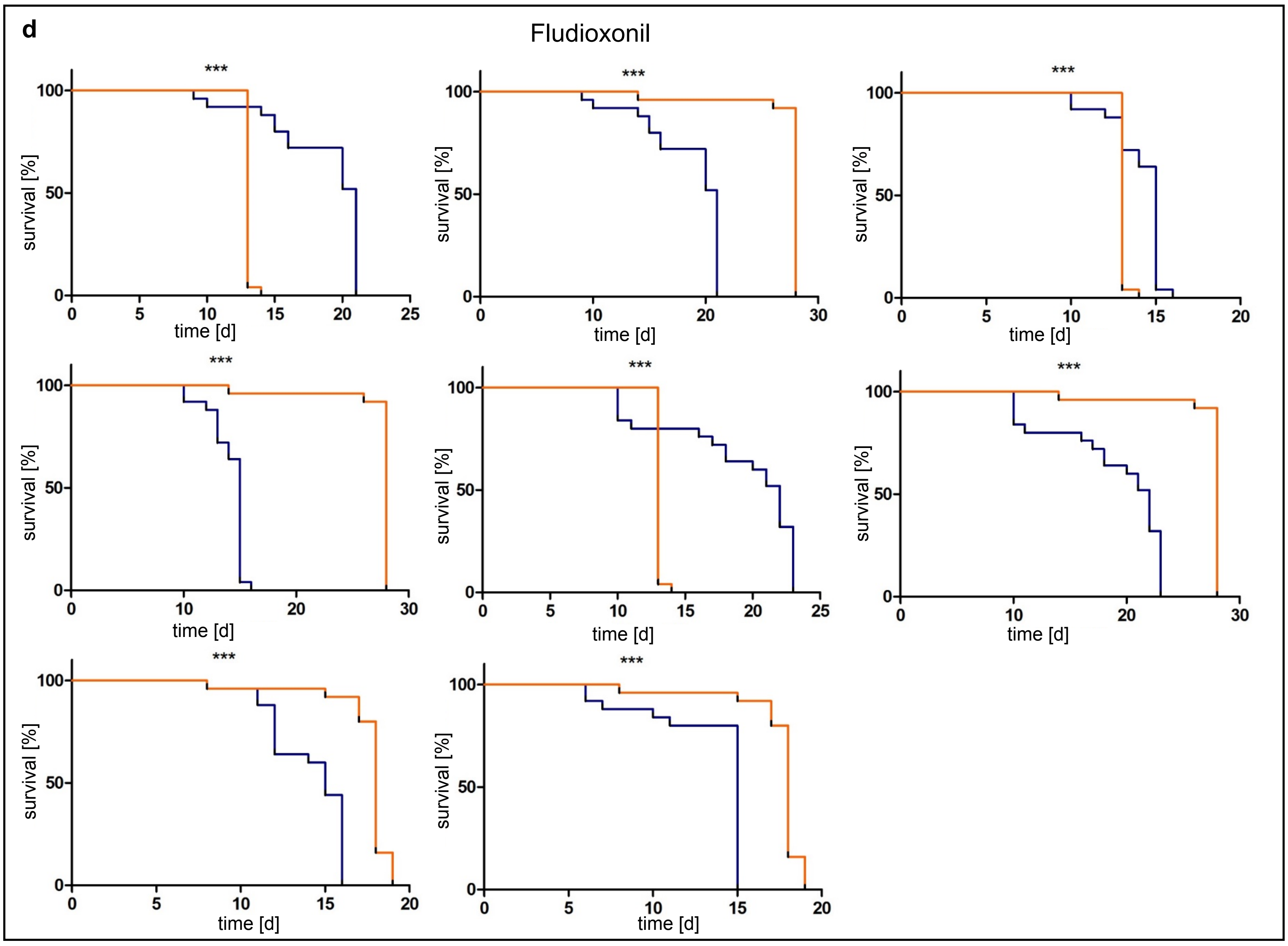

Exposure to low doses of pesticides induces an immune response and the production of nitric oxide in honeybees

Merle T. Bartling[a], Susanne Thümecke[a], José Herrera Russert[a], Andreas Vilcinskas[a,b], Kwang-Zin Lee[b,1]

[a]Institute for Insect Biotechnology, Justus Liebig University of Giessen, Heinrich Buff Ring 26-32, D−35392, Germany
[b]Fraunhofer Institute for Molecular Biology and Applied Ecology, Ohlebergsweg 12, D-35394 Giessen, Germany

[1]To whom correspondence may be addressed. Email kwang-zin.lee@ime.fraunhofer.de

Figure S1

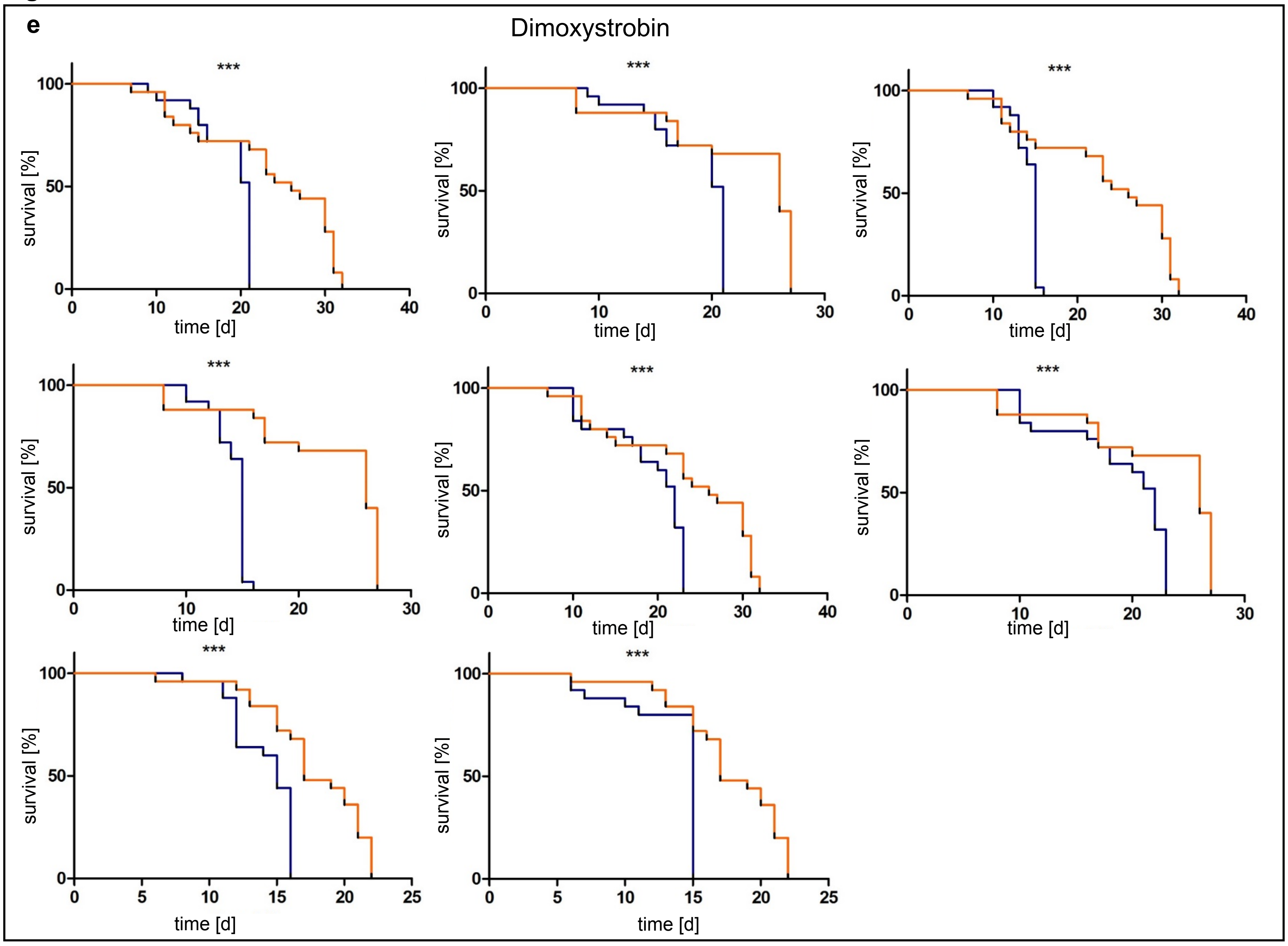

Supplement: Supplementary file 1 — Supplementary Information 1. [file 41598_2021_86293_MOESM1_ESM.pdf]
