# Supplementary material for: Exposure to low doses of pesticides induces an immune response and the production of nitric oxide in honeybees
Source: Sci Rep. 2021 Mar 25;11:6819. doi: 10.1038/s41598-021-86293-0 (PMC7994568; doi:10.1038/s41598-021-86293-0)
Supplement: Supplementary file 2 — Supplementary Information 2. [file 41598_2021_86293_MOESM2_ESM.docx]

**Exposure to low doses of pesticides induces an immune response and the production of nitric oxide in honeybees**

Merle T. Bartling^a^, Susanne Thümecke^a^, José Herrera Russert^a^, Andreas Vilcinskas^a,b^, Kwang-Zin Lee^b,1^

^a^Institute for Insect Biotechnology, Justus Liebig University of Giessen, Heinrich Buff Ring 26-32, D‑35392, Germany

^b^Fraunhofer Institute for Molecular Biology and Applied Ecology, Ohlebergsweg 12, D-35394 Giessen, Germany

^1^To whom correspondence may be addressed. Email kwang-zin.lee@ime.fraunhofer.de

**Supplementary Information**

**Table S1** Primers used for quantitative real-time RT-PCR.

| **Target** | **Accession number** | **Forward** | **Reverse** |
| --- | --- | --- | --- |
| *eIF3-S8* | XM_006564593.3 | AAGAGAGTTGTACGATCAACCA | TCTTCAAAACTGGACAACATGC |
| *apisimin* | XM_017065401 | GTCGTCCTAGCTGCCTTCTG | GGAAACGACATCCACGTTCG |
| *defensin-1* | NM_001011616 | GGCTGCACCTGTTGAGGAT | TGTCCTTTGAATGAGAGAAGGTCA |
| *-hymenoptaecin* | NM_001011615 | ACAATGGATTATATCCCGACTCGT | CAATGTCCAAGGATGGACGAC |
| *defensin-2* | NM_001011638 | TGCGACGTTTTATCATGGCA | CTACCGCCTTTACGTCGTTG |
| *abaecin* | AF442147 | ATGCGCAGCATTCGCATACG | AATTTTCGGATTGAATGGTC |
| *cactus-2* | XM_394485 | TGTGGAGCTCGTACGACTTT | TAGCGAGATGAAGAGCCGTT |
| *cyp9e2* | KT697624 | GTAGGGAGAAGTTGGGCACC | GCTACGTTCTCGACGGTCAT |
| *cyp6a17* | XM_395671.6 | TCGTTTTGCGCGATCTTGAC | AAAAAGGTTCGGCTCGTTCG |
| *cyp6k1* | XM_006564436.3 | TGGCGGATTACAAGATACCAGG | AACGGAGAGGATCTGGATGA |
| *cyp314a1* | XM_026440529 | CGTAACTCCAGGTATCCGCT | GTCAATTCCGACGTGAGAGC |
| *cyp6as5* | NM_001040234.1 | TGCCGTATTGTTTCTTGCCC | TGGTACAGGTTTCGGTCCAA |
| *UGT-2C1* | XM_026441208 | AAGTGTGCTCGCTCATCCAA | GGTATACCGAGAACTGGTACTTCA |
| *UGT-2B15* | XM_016912925 | CGCGGGGCTATCTTTTCTTC | TGATGGCTATAAGACGGCGT |
| *hopscotch* | XM_006567689 | TCACAGAGCGATTTGGAGGT | ACCATTTTCCAGCAAGTTCCA |
| *NOS* | NM_001012962 | TCCACTCGCAGGTACTTTCC | TCTGGAGGATCACCATTTCC |
| *duox* | XM_026439464.1 | CCCTAATAGCCCTCGTGAAG | GAGCATTCTCATAACGTGTG |
| *catalase* | NM_001178069 | GTCTTGGCCCAAACAATCTG | CATTCTCTAGGCCCACCAAA |

**Table S2** Conditions used for quantitative real-time RT-PCR.

| **Description** | **Temperature** | **Time** |
| --- | --- | --- |
| **Holding stage** | **Step 1** |  |
|  | 95°C | 10 min |
| **Cycling stage** | **40 cycles** |  |
|  | **Step 1** |  |
|  | 95°C | 15 sec |
|  | **Step 2** | 90 sec |
|  | 60°C | 60 sec |
| **Melt curve stage** | **Step 1** |  |
|  | 95°C | 15 sec |
|  | **Step 2** |  |
|  | 60°C | 60 sec |
|  | **Step 3** |  |
|  | 95°C | 15 sec |
| **Temperature increment**  **(melt curve)** | 0.5°C |  |
